# Advances in Autism Research: Series II

**DOI:** 10.3390/brainsci13020332

**Published:** 2023-02-15

**Authors:** Antonio Narzisi

**Affiliations:** IRCCS Stella Maris Foundation, 56018 Pisa, Italy; antonio.narzisi@fsm.unipi.it

“Advances in Autism Research: Series II” is a continuation of the important Special Issue (SI) published in 2020 that collected 50 articles [1].

In “Advances in Autism Research: Series II”, eight articles, between 2020 and 2021, took part in this SI. Globally, all articles involved 59 authors and 16 reviewers between researchers and clinicians actively engaged in the field of Autism Spectrum Disorder (ASD) and Neurodevelopmental Disorders.

Devescovi and colleagues [2] at the Institute for Maternal and Child Health—IRCCS “Burlo Garofolo” in Trieste (Italy) evaluated the effectiveness of a low-intensity Early Start Denver Model (ESDM) program delivered in the Italian community. They compared a cohort of children aged 19 to 43 months, who received ESDM for 2 h per week for one year, with a concurrent non-randomized control group receiving standard care (Treatment as Usual-TAU). Both groups underwent evaluation at the start of the study (T0), and after 6 months (T1) and 12 months (T2) of intervention. Despite similar advancements in cognitive and linguistic abilities, the group receiving ESDM demonstrated greater improvement in communication, social skills, and maladaptive behavior, as measured by the ESDM Curriculum Checklist. This study supports the feasibility of ESDM implementation within the Italian public health system and suggests a more favorable outcome for the group receiving ESDM compared to the control group.

Marino and his team [3] at the National Research Council of Italy (CNR) assessed the effectiveness of the Acceptance Commitment Therapy (ACT) protocol in comparison to the Parent Training (PT) program. A group of twelve parents were divided into two demographically similar groups and underwent 24 weekly sessions of either the ACT protocol (experimental group) or the traditional PT (control group). Results indicated that parents participating in the ACT protocol displayed significant advancements in psychological flexibility, consciousness, and personal values in daily life, and reduced parental stress. Meanwhile, there was a decrease in the parents’ views of their child’s misbehavior.

The Abou-Abbas study [4] at McGill University in Montreal, Canada, introduced a new methodology for categorizing visual event-related potentials (ERPs) data gathered from 6-month-old infants. The technique employs intrinsic mode functions (IMFs) obtained from empirical mode decomposition (EMD). These IMFs were used as inputs to two machine learning techniques (support vector machines and k-nearest neighbors (k-NN)) with nested cross-validation. The study examined the risk classification of both control groups and high-risk (HR) groups and the diagnostic outcome within the high-risk group: HR-ASD and HR-noASD. The highest accuracy rate for classifying familial risk was achieved using a support vector machine (SVM) with 88.44%. The highest accuracy rate for differentiating infants at risk who developed ASD from those who did not was 74.00% using k-NN. The IMF-based features demonstrated remarkable efficiency in determining infant risk status but were not as effective in diagnosis outcome classification. This advanced signal analysis of ERPs in combination with machine learning could be considered a preliminary step in creating an early biomarker for ASD.

Cai and his colleagues [5] at Yangzhou University (China) assessed the effects of a prolonged exercise intervention on social cognition (SC) and white matter integrity (WMI) in children diagnosed with autism and further explored the neural effect of exercise on SC. A total of 29 children aged 3 to 6 with autism were divided into two groups, an exercise group (15 participants) and a control group (14 participants). The exercise group underwent a 12-week mini-basketball training program (five sessions per week, 40 min each), while the control group was instructed to continue their daily activities. Both groups were evaluated before and after the intervention for SC and WMI. Results showed that the SC scores were lower in the exercise group post-intervention. The WMI of the exercise group displayed higher fractional anisotropy in multiple brain regions and reduced mean diffusivity in some regions when compared to the control group. Additionally, a correlation was found between elevated WMI and lower scores on a social cognition measure for the entire sample. This study presents the first evidence that exercise intervention improves SC and WMI in children with autism.

The research conducted by Rahman and colleagues [6], located at the Faculty of Biological Sciences at the Islamic University in Bangladesh, aimed to profile the gene expression patterns of the brain cortex in individuals with autism spectrum disorder (ASD). The study utilized two publicly available RNA-seq studies to discover new genes related to ASD through a meta-analysis. The results showed that there were 1567 differentially expressed genes (DEGs), with 1194 being upregulated and 373 being downregulated. The analysis also uncovered 235 novel DEGs not detected in the original RNA-seq studies, including seven genes (PAK1, DNAH17, DOCK8, DAPP1, PCDHAC2, ERBIN, and SLC7A7) previously linked to ASD. The study revealed that the DEGs were enriched in several molecular pathways, including osteoclast differentiation, the TNF signaling pathway, and the complement and coagulation cascade. The protein–protein interaction network analysis revealed several proteomics hub gene signatures, including MYC, TP53, HDAC1, CDK2, BAG3, CDKN1A, GABARAPL1, EZH2, VIM, and TRAF1, and the transcriptional factors regulating the DEGs were determined to be FOXC1, GATA2, YY1, FOXL1, USF2, NFIC, NFKB1, E2F1, TFAP2A, and HINFP.

The review by Podgórska-Bednarz and Perenc [7], conducted at the Medical College of Rzeszow University in Poland, aimed to assess the therapeutic benefits of Hyperbaric Oxygen Therapy (HBOT) for individuals with autism spectrum disorder (ASD). Out of 10 literature reviews evaluated, none confirmed that HBOT is an effective form of treatment for ASD. Of the four studies that investigated the effects of HBOT on ASD, two found no support for its use and the results of the other two studies were not conclusive, as one lacked a control group and the other only focused on auditory processing disorders. The overall review of the literature on HBOT and its impact on the symptoms of ASD did not provide evidence of its effectiveness.

The aim of the investigation conducted by Carmona-Serrano and colleagues [8] at the University of Granada (Spain), was to monitor the development of scientific literature regarding autism and the utilization of technology in treating this disorder [9]. A bibliometric method was applied, based on a co-word analysis, utilizing the Web of Science database. The analysis was conducted on 1048 publications with the aid of the SciMAT software. The findings indicate that the initial studies on the subject emerged in 1992, but it wasn’t until 2009 that the volume of research increased significantly. The majority of the studies were centered on rehabilitation, emphasizing the therapeutic aspect of the research. The results show that the focus of the research was mainly on interventions carried out using technological resources for individuals with autism, particularly students and young people.

In the study by Narzisi and Muccio [10] at the Stella Maris Scientific Institute (Italy), the authors present a unique perspective on autism spectrum disorder (ASD) that departs from the traditional focus on relational issues and instead draws from philosophy, specifically Husserlian phenomenology. The authors start by examining recent etiological viewpoints that posit a natural tendency towards hypersensitivity and reduced cognitive priors in some individuals with ASD. These two qualities are viewed as a kind of phenomenological a priori that could make individuals with ASD more prone to spiritual experiences, understood as conscious awareness and attention to the present moment, rather than in a religious sense. The authors explore the potential clinical implications of this perspective.
